# 3D Seed-Germination-Like MXene with In Situ Growing CNTs/Ni Heterojunction for Enhanced Microwave Absorption via Polarization and Magnetization

**DOI:** 10.1007/s40820-021-00680-w

**Published:** 2021-07-19

**Authors:** Xiao Li, Wenbin You, Chunyang Xu, Lei Wang, Liting Yang, Yuesheng Li, Renchao Che

**Affiliations:** 1 Laboratory of Advanced Materials, Shanghai Key Lab of Molecular Catalysis and Innovative Materials, Fudan University, Shanghai, 200438 People’s Republic of China; 2 Department of Materials Science, Fudan University, Shanghai, 200438 People’s Republic of China

**Keywords:** Microwave absorption, Two-dimensional materials, MXene, Magnetic coupling network, Synergistic effect

## Abstract

- Benefiting from the possible “seed-germination” effect, the “seeds” Ni^2+^ grow into “buds” Ni nanoparticles and “stem” carbon nanotubes (CNTs) from the enlarged “soil” of MXene skeleton.
- Compared with the traditional magnetic agglomeration, the MXene-CNTs/Ni hybrids exhibit the highly spatial dispersed magnetic architecture.
- 3D MXene-CNTs/Ni composites hold excellent microwave absorption performance (−56.4 dB at only 2.4 mm).

**Abstract:**

Ti_3_C_2_T_x_ MXene is widely regarded as a potential microwave absorber due to its dielectric multi-layered structure. However, missing magnetic loss capability of pure MXene leads to the unmatched electromagnetic parameters and unsatisfied impedance matching condition. Herein, with the inspiration from dielectric-magnetic synergy, this obstruction is solved by fabricating magnetic CNTs/Ni hetero-structure decorated MXene substrate via a facile in situ induced growth method. Ni^2+^ ions are successfully attached on the surface and interlamination of each MXene unit by intensive electrostatic adsorption. Benefiting from the possible “seed-germination” effect, the “seeds” Ni^2+^ grow into “buds” Ni nanoparticles and “stem” carbon nanotubes (CNTs) from the enlarged “soil” of MXene skeleton. Due to the improved impedance matching condition, the MXene-CNTs/Ni hybrid holds a superior microwave absorption performance of − 56.4 dB at only 2.4 mm thickness. Such a distinctive 3D architecture endows the hybrids: (i) a large-scale 3D magnetic coupling network in each dielectric unit that leading to the enhanced magnetic loss capability, (ii) a massive multi-heterojunction interface structure that resulting in the reinforced polarization loss capability, confirmed by the off-axis electron holography. These outstanding results provide novel ideas for developing magnetic MXene-based absorbers. 
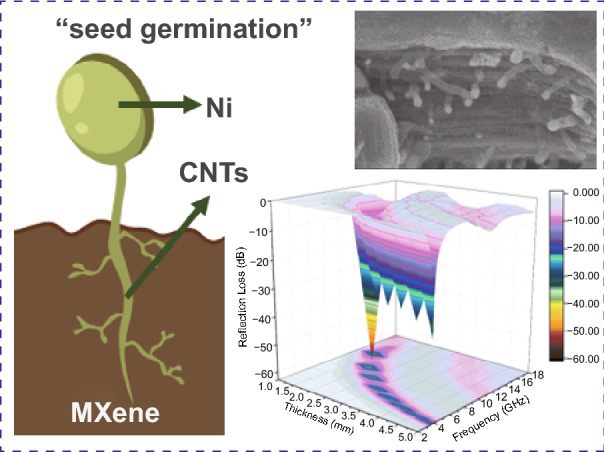

**Supplementary Information:**

The online version contains supplementary material available at 10.1007/s40820-021-00680-w.

## Introduction

Microwave absorption (MA) materials are highly demanded in many areas of 5G high-frequency communication, wireless systems, military stealth, and prevention of electromagnetic (EM) wave interference & pollution [1–5]. Conventionally, a superior MA absorber depends mainly on a satisfied impedance condition (*Z* value) and a distinct EM attenuate capability (*α* value) that composed of polarization [6, 7], conductive [8, 9], and magnetic loss [10, 11]. Well-matched *Z* value indicates that as much EM wave as possible enters the interior of material to reduce its reflection on the surface, which is the precondition for the subsequent EM dissipation [12]. According to the *Z* formula

1 $$Z = \left| {\frac{{Z_{{{\text{in}}}} }}{{Z_{0} }}} \right| = \sqrt {\left| {\frac{{\mu _{r} }}{{\varepsilon _{r} }}} \right|} \tanh \left[ {j\left( {\frac{{2\pi fd}}{c}} \right)\sqrt {\mu _{r} \varepsilon _{r} } } \right]$$

excessively high conductive loss could lead to an unbalanced impedance matching condition [13], resulting in the decreased MA performance [14]. Hence, the principle of designing an excellent MA absorber is to balance the *Z* value and *α* value accompany by improving its polarization loss and magnetic loss rather than conductive loss [15].

Recently, owing to the advantages of unique layered structure, tunable active surface and outstanding electrical conductivity, 2D MXene materials have aroused considerable research interests [16–20]. The various etching conditions lead to the formation of MXene with different morphologies (single-layered with high conductivity and multi-layered with low conductivity). Notably, proper conductivity is better for MA materials rather than the highest one. Most of the reported MXene-based composites are based on the multi-layered MXene. Nevertheless, limitations such as low dielectric loss and absent magnetic loss have greatly restricted their overall MA performance (only − 11 dB) [21]. Two typical methods have generally been used to overcome these drawbacks. One is to introduce high dielectric loss materials to compound MXene via the heterogeneous interfacial construction, such as surface decoration [22], element doping [23], and micro–nano architecture designing [24]. Owing to the excellent conductivity, light weight and good environment stability, carbon (C) materials, mainly including C nanospheres [25], 1D CNTs [26], 2D graphene oxide (GO/rGO) [27], and 3D graphene foam [2], are the common high dielectric loss materials. Zhao et al. prepared MXene/C nanospheres hybrids with massive heterogeneous interface, indicating the reflection loss (RL) value of − 54.67 dB [25]. Yin et al. reported that the MA performance of MXene/CNTs composites was improved to − 52.9 dB [26]. However, the effective absorption band (EAB, the bandwidth of RL ≤ − 10 dB) of these composites are slightly narrower because of the absence of magnetic loss capability. Considering the importance of dielectric-magnetic complementarities, introducing magnetic nanoparticles (e. g., Ni [28], Fe_3_O_4_ [29], and CoFe [30]) into the dielectric MXene skeleton has emerged as another effective way to improve the MA performances. Liang et al. fabricated MXene/Ni chain composites through the simple physical mixing (RL = −  49.9 dB) [31]. Yan et al. decorated MXene with FeCo nanoparticles to reduce its excessive complex permittivity [32]. Nevertheless, the problem of magnetic agglomeration and uneven distribution is unavoidable. Therefore, combining the advantages of carbon materials and magnetic particles to fabricate the well-organized ternary microstructures may be a viable solution to overcome their respective shortcomings.

It is widely accepted that a reasonable electromagnetic recombination method plays a key factor in the final MA performance [33]. Liu et al*.* used simple blending method to compound MXene and Ni chain [31]. However, the growth of dielectric/magnetic units tend to trigger uneven dispersed distribution and adverse local matching imbalance, resulting in the precarious of the MA performance. In addition, the special multi-layered structure of accordion-like MXene further limits the size and distribution of magnetic nanoparticles. The narrow layer spacing prevents magnetic nanoparticles from entering the MXene layer. Notably, using the connection points of the hollow 1D CNTs and its confinement effect [4], the magnetic nanoparticles located inside can be fully dispersed, thereby inhibiting the agglomeration phenomenon. However, how to simultaneously obtain uniform polarization loss, conductive loss, and magnetic loss is still a considerable challenge.

Herein, a distinct ternary 3D MXene-CNTs/Ni hybrid with encouraging MA property is successfully prepared. The hybrid owns the typical seed-germination-like morphology: the multi-layered MXene act as the enlarged “soil,” numerous Ni^2+^ ions serve as the “seeds” to germinate and then become the “buds” Ni nanoparticles that embedded in the top of “stem” CNTs. Thus, the MXene-CNTs/Ni composite holds the significantly enhanced MA performance (− 56.4 dB) at only 2.4 mm thickness. Compared with the traditional magnetic agglomeration, the MXene-CNTs/Ni hybrids own the highly spatial dispersed magnetic architecture. The suspended Ni nanoparticles in massive orientated CNTs framework can form 3D magnetic coupling network. Moreover, this magnetized MXene composite achieves the improved impedance matching condition. This work may enlighten the design of other MXene-based materials.

## Experimental Section

### Materials

Ti_3_AlC_2_ MAX was purchased from Jilin 11 Technology Co., Ltd. Nickel chloride (NiCl_2_·6H_2_O), melamine (C_3_N_3_(NH_2_)_3_), lithium hydroxide (LiOH), and ethanol were purchased from Sinopharm Chemical Reagent Co., Ltd.

### Preparation of MXene

The MAX powder was put in 40% HF solutions for 24 h to remove Al layers. Afterward, the black product was cleaned with deionized water several times (pH ≈ 6–7). Finally, the as-prepared MXene was collected in the freeze dryer at − 40 °C for 24 h.

### Preparation of MXene-alk

MXene was immersed in 1 M LiOH solution for 24 h to expand the interlayer spacing. Afterward, the black product was rinsed with deionized water several times (pH ≈ 7–8). The obtained MXene-alk was vacuum dried at 80 °C for 12 h.

### Preparation of CNTs/Ni

About 0.5 g NiCl_2_·6H_2_O and 3 g melamine powders were fully mixed by grind in a mortar. Afterward, the sample was heated to 800 °C for 2 h under N_2_ atmosphere. Finally, the black products were obtained and denoted as CNTs/Ni.

### Preparation of MXene-N

For comparison, the as-prepared MXene was heated to 800 °C for 2 h under N_2_ atmosphere. Finally, the black products were obtained and denoted as MXene-N.

### Preparation of MXene-CNTs/Ni

The as-prepared MXene was immersed in 1 M LiOH solution for 24 h to expand the interlayer spacing. Then the NiCl_2_·6H_2_O and melamine powders (30 wt%) were added to the MXene solution and mix well during stirring. After the drying treatment, the sample was further heated to 800 °C for 2 h under N_2_ atmosphere. Finally, the black products were obtained and denoted as MXene-CNTs/Ni.

### Characterization

All as-prepared samples were characterized by an X-ray powder diffraction (XRD) with Ni-filtered Cu K*α* radiation (40 kV, 40 mA) that operated by a D8 ADVANCE X-ray diffractometer (Bruker), scanning electron microscopy (SEM) that worked with Hitachi S-4800 field-emission scanning electron microscope, transmission electron microscopy (TEM), selected-area electron diffraction (SAED), high resolution TEM (HRTEM), and off-axis electron holography measurements were used with a JEM-2100F transmission electron microscope. The EM parameters of all as-prepared samples were analyzed by a N5230C vector network analyzer. The as-prepared samples were fabricated by uniformly mixed with paraffin matric (mass fraction of 30 wt%) and further pressed into a coaxial ring with an outer diameter of 7 mm and an inner diameter of 3.04 mm. The RL value were calculated by the following equations [29, 30]:

2 $$Z = \left| {Z_{{{\text{in}}}} /Z_{0} } \right| = \sqrt {\mu _{r} /\varepsilon _{r} } \tanh [j(2\pi fd/c)\sqrt {\varepsilon _{r} \mu _{r} } ]$$

3 $${\text{RL}}\left( {{\text{dB}}} \right) = 20\lg \left| {(Z_{{{\text{in}}}} - Z_{0} )/\left( {Z_{{{\text{in}}}} + Z_{0} } \right)} \right|,$$

where *μ*_*r*_ ($\mu _{r} = \mu ^{\prime} - j\mu ^{\prime\prime}$) is the complex permittivity, *ε*_*r*_ ($\varepsilon _{r} = \varepsilon ^{\prime} - j\varepsilon ^{\prime\prime}$) is the complex permeability, *Z*_in_ is the input impedance, *Z*_0_ is the impedance of free space, *f* is the frequency of incident EM wave, *d* is the thickness, and *c* is the light velocity.

## Results and Discussion

### Fabrication and Characterization of MXene-CNTs/Ni Composites

**Scheme 1:**
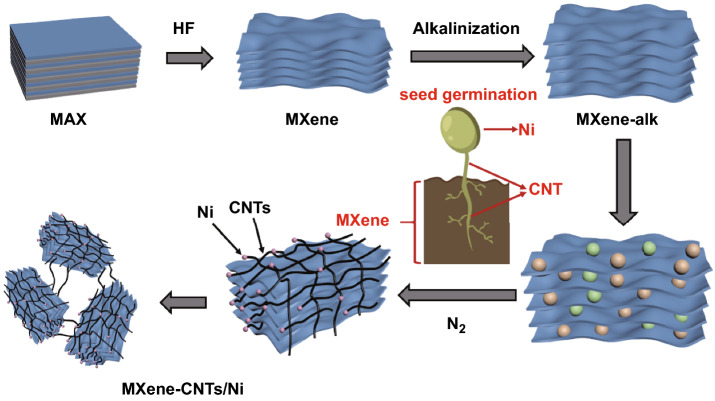
Schematic preparation process of 3D MXene-CNTs/Ni composite

The main process of preparing MXene-CNTs/Ni composites is described in Scheme 1. Firstly, the MAX powder is immersed in 40% HF for 24 h to selectively remove the Al layers. Secondly, the obtained MXene is immersed in LiOH solution, which can effectively expand interlayer spacing of MXene [34]. Thirdly, the alkalized MXene is putted in NiCl_2_ and melamine solution to mix well during stirring [35]. Ni^2+^ ions could attach at both surface and interlamination of each multi-layered MXene unit due to the ion exchange and electrostatic interaction [36]. Fourthly, melamine provides a carbon source for subsequent reactions. Nickel chloride not only acts as a nickel source, but also provides catalytically active sites. In the pyrolysis process, melamine is first decomposed into carbon nitride nanosheet structure. Then, the nickel ions are converted into metallic nickel nanoparticle catalysts accompanied by a fluid nitrogen atmosphere. Under the catalysis of the Ni nanoparticles, the carbon nitride nanostructures are decomposed into C–N groups and rearranged to form in situ bamboo-shaped graphitic CNTs [37, 38]. Finally, the typical bamboo-like CNTs in which Ni particles are embedded on the top are formed in the surface and interlamination of each accordion-like MXene unit. Meanwhile, the CNTs on adjacent MXene units are gradually connected to construct the mono-dispersed MXene units into a whole network.

**Fig. 1:**
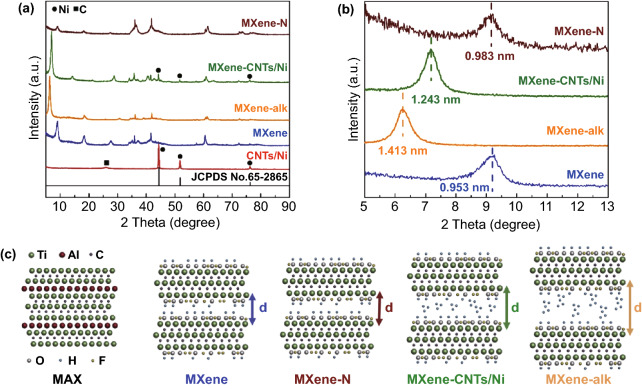
**a** XRD patterns of four different samples (MXene, MXene-alk, MXene-N, and MXene-CNTs/Ni). **b** Magnification of XRD patterns in a. **c** Layer spacing model of four different samples (MXene, MXene-alk, MXene-N, and MXene-CNTs/Ni)

The XRD patterns of MAX and MXene are illustrated in Fig. S1. All intensive diffraction peaks of the raw powder are matched well with the MAX (JCPDS No. 52-0875) [39]. After the intensive HF exfoliation, the main peak (002) of MXene is clearly shifted to the lower angles with the higher *c* parameters. It is worth noting that the crystallinity of MXene is slightly lowered compared to that of MAX. Figure 1a indicates the XRD patterns of the MXene, MXene-alk, MXene-N, and MXene-CNTs/Ni samples. The corresponding detailed range from 5 to 13 degrees is presented in Fig. 1b. Compared to the XRD patterns of MXene, the main peak (002) of other three samples are shifted to lower angles. The corresponding interlayer spacing is increased from 0.953 to 1.413 nm. The radius of Ni^2+^ (0.069 nm) is clearly smaller than the interlayer spacing of MXene-alk (1.413 nm), indicating the possibility of Ni^2+^ ions intercalation. After heat treatment under N_2_ atmosphere, the Ni^2+^ ions are reduced to Ni nanoparticles, which in turn acts as a catalyst to benefit the in situ formation of CNTs. Since the sizes of CNTs and Ni are much larger than 1.413 nm, some CNTs/Ni composites in situ grow between the layers of multi-layered MXene substrate could slightly reduce the average interlayer spacing of MXene-CNTs/Ni to 1.243 nm. Remarkably, the peak at 2θ = 26.5°, 44.5°, 51.8°, 76.4° are presented in the CNTs/Ni and MXene-CNTs/Ni, proving the successfully preparation of CNTs (JCPDS No. 65-6212) and Ni (JCPDS No. 65-2865) [40].

**Fig. 2:**
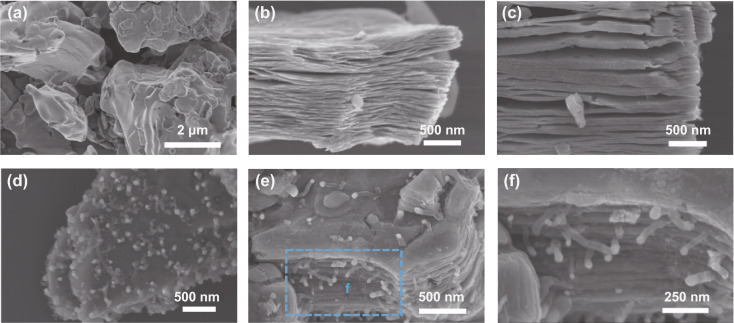
SEM images of **a** MAX, **b** MXene, **c** MXene-alk, and **d–f** MXene-CNTs/Ni

**Fig. 3:**
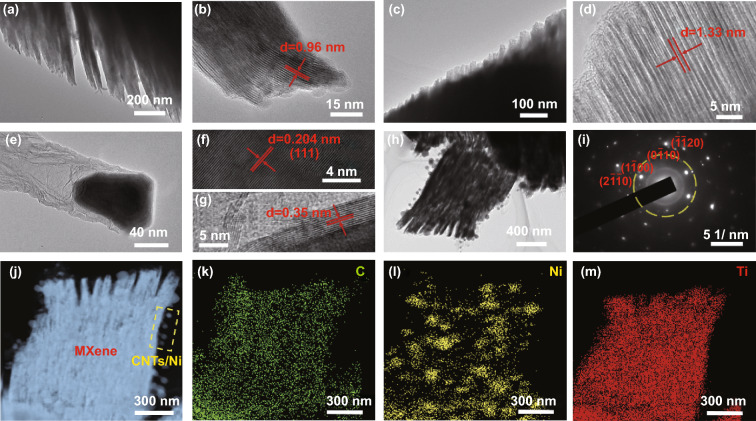
TEM images of **a** MXene, **c** MXene-alk, **e** CNTs/Ni and **h** MXene-CNTs/Ni composites. HRTEM images of **b** MXene, **d** MXene-alk, **f** Ni and **g** CNTs. STEM images of **j–m** MXene-CNTs/Ni and corresponding elemental mapping of C, Ni, and Ti

The SEM images of MAX before and after the strongly HF etching process are illustrated in Fig. 2a, b, where the nanosheets are obviously separated from each other compared to the raw sample. As previously reported, the MXene have the typical accordion-like multi-layered microstructure. Figure S2a, c show the morphology of MXene-N and MXene-alk, respectively, which maintains a similar multi-layered architecture compared to the pristine MXene. This shows that the high-temperature calcination and alkalization treatment did not destroy the original morphology of MXene. SEM image of CNTs/Ni is shown in Fig. S3a. Clearly, the Ni nanoparticles are embedded at the top of the relatively straight and microns-length CNTs. As shown in Fig. 2d–f, CNTs/Ni are uniformly dispersed and growth in the surface and interlamination of the MXene units. Due to the confinement effect of the accordion-like MXene, the length of CNTs in the MXene-CNTs/Ni composite is significantly shorter compared to that of pure CNTs/Ni sample. Thanks to the part of Ni^2+^ ions are intercalated between the MXene layers, these carbon nanotubes grow from the interlaminations and further interweave together. The TEM image in Fig. 3a shows the multi-layered structure of MXene after intensive HF treatment, indicating the successful exfoliation. This morphology is matched with the SEM image in Fig. 2b. The cross-sectional HRTEM image of MXene obviously show that the interlayer spacing is about 0.96 nm (Fig. 3b), in good accordance with the XRD result in Fig. 1b. Both MXene-N and MXene-alk maintain their original multi-layered structure. Importantly, the interlayer spacing of MXene-alk is increased to 1.33 nm after the alkalization treatment (Fig. 3c, d). CNTs/Ni sample exhibits the typical bamboo-like structures with the Ni nanoparticles that embedded in the connect joints (Figs. 3e and S3b–d), supporting the growth mechanism of the oriented CNTs. Figures 3f, g indicates the HRTEM images of CNTs/Ni. Ni nanoparticle is tightly enwrapped by the carbon layers, representing the well-connected heterogeneous interfaces. Ni nanoparticle shows the clear lattice fringes, revealing the distinct crystallinity. The interplanar spacing is 0.204, corresponding to the (111) plane of *fcc* Ni. The Ni nanoparticle with ~ 100 nm diameter are surrounded by a few carbon layers with the thickness of 0.35 nm. As shown the TEM image in Fig. 3h, the tangled CNTs are homogeneously high-density distributed in the surface and interlaminations of MXene unit, revealing the formation of the 3D microstructure. The corresponding SAED pattern of MXene-CNTs/Ni composite composed of a hexagonal lattice of MXene and the CNTs/Ni diffraction rings with low concentration (Fig. 3i). A clear multi-layered structure can be described from the STEM image of Ni^2+^-MXene-alk sample (Fig. S4). The uniform high-dispersed distribution of the Ni^2+^ ions on the framework can be illustrated by the EDS elemental maps of Ni (Fig. S4b–d). Differently, there are obvious aggregation points in the elemental maps of Ni distribution in MXene-CNTs/Ni (Fig. 3j–m), which can be concluded by combining with the SEM images (Fig. 2e) that the Ni nanoparticles are successfully synthesized. As shown in the Ni 2p spectrum (Fig. S5), peaks at 854.5 and 872.3 eV are belonging to Ni^0^ in Ni 2p_3/2_ and Ni 2p_1/2_, respectively. Peaks at 856.3 and 875.2 eV are ascribed to Ni^0^ in Ni 2p_3/2_ and Ni 2p_1/2_, indicating the successful synthesis of Ni nanoparticles [41]. Moreover, the C 1 s spectra of MXene-CNTs/Ni shows three peaks at 281.9, 284.6, and 285.7 eV corresponding to C–Ti, C–C and C–O bonds, respectively (Fig. S6a). The Ti 2p spectra of MXene-CNTs/Ni can be divided into many subpeaks corresponding to the Ti–C, Ti–O, Ti (II) signals, respectively. The existence of Ti–O bond can be attributed to the small part of the oxidation of the Ti layer during the calcination process.

### Electromagnetic Parameters Analysis and Microwave Absorption Performance

**Fig. 4:**
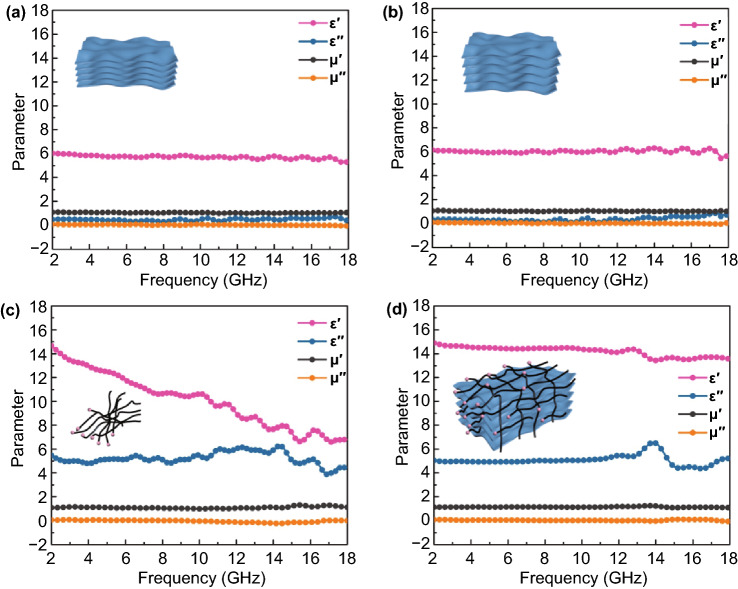
Permittivity and permeability versus frequency of **a** MXene, **b** MXene-N, **c** CNTs/Ni and **d** MXene-CNTs/Ni composites

The electromagnetic parameters of MXene, MXene-N, CNTs/Ni, and MXene-CNTs/Ni samples are plotted in Fig. 4 to construct the relationship between microstructure and MA performance. For pure MXene, the average εʹ and εʹʹ values are 5.71 and 0.46 respectively, implying the low dielectric loss capability. The average εʹ value of MXene-N shows a slightly increasing tendency and reached to 6.04, while the εʹʹ value is basically maintained at 0.35. This slight growth tendency is mainly attributed to the presence of some amorphous carbon during the heat treatment. Notably, both εʹ and εʹʹ values of MXene and MXene-N are close, indicating that the nanometer widening of the MXene layer spacing does not have a significant impact on dielectric loss capability. As expected, the εʹ and εʹʹ values obviously rise to the range of 14.89–13.57 and 6.54–4.38 in MXene-CNTs/Ni composites, respectively. In principle, the εʹ is mainly attributed to the polarization, while the εʹʹ is mainly determined by the conductivity of samples [42]. Therefore, the increase in εʹ of MXene-CNTs/Ni is attributed to the enlarged heterojunction interfaces and the associated increased polarization behavior, while the growth trend of εʹʹ is due to the formation of 3D conductive network after the introduction of CNTs with high conductivity loss. Both MXene and MXene-N unable to produce magnetic loss due to its inherent dielectric property, in which μʹ and μʹʹ values are close to 1 and 0, respectively [43]. Comparatively, the other two Ni-based samples show a significantly improved complex permeability. The bamboo-like CNTs with massive connect joints have the advantage in inhibiting magnetic imbalanced distribution, successfully increasing the effective use of the Ni nanoparticles with maximized magnetic surface. Meanwhile, the curled CNTs also connect each independent magnetized MXene unit, further forming a 3D magnetic coupling network. The above-mentioned results obviously demonstrated that the in situ growth of CNTs/Ni as a dielectric/magnetic modifier optimized the EM parameters in that of unsatisfied MXene, which is the critical factor to further improve the MA performance.

**Fig. 5:**
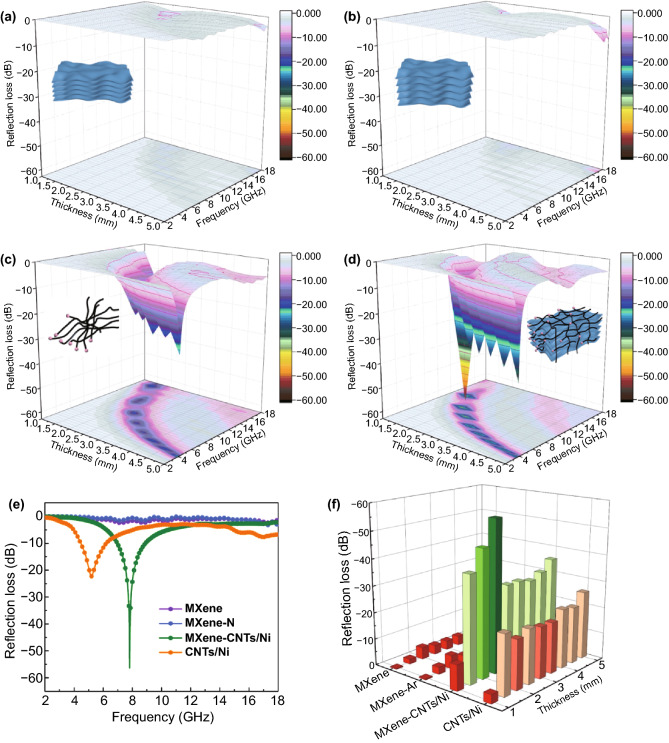
3D plots for **a** MXene, **b** MXene-N, **c** CNTs/Ni and **d** MXene-CNTs/Ni composites. **e** Compared RL curves and **f** calculated 3D bars for four different samples

Generally, the MA performance is main directly evaluated by the RL and EAB values. The RL values versus thickness of MXene, MXene-N, CNTs/Ni, and MXene-CNTs/Ni are calculated in Fig. 5. Both MXene and MXene-N show the negligible MA performance due to the weak dielectric loss and lack magnetic loss capability. The CNTs/Ni demonstrates the middle level of MA property accompanied with a RL value of − 22.3 dB at 5.2 GHz, which can be attribute to the excessive permittivity and unsatisfied impedance. Comparatively, the MXene-CNTs/Ni hybrid holds the best MA property with a RL value of as high as − 56.4 dB at only 2.4 mm. When the thickness is adjusted to 1.5 mm, its EAB can reach 3.95 GHz. These data exceed other reported MXene/CNT-based MA absorber previously (Table S1). Moreover, the MA performance of MXene/Ni/CNTs obtained by physically mixing MXene/Ni and CNTs have been tested (Fig. S7). Compared with the MXene/CNTs/Ni (− 28.7 dB, 14.64 GHz), the synthesized MXene-Ni/CNTs shows superior MA performance. This finding demonstrates that the traditional magnetic agglomeration induced by simple mixture can be effectively avoided by the distinctive 3D architecture of MXene-Ni/CNTs, and consequently leading to the enhanced MA performance. In addition, the MA performance of synthesized MXene-Ni/CNTs composite shows a better reflection loss than that of MXene/Ni composite. The results indicate that the presence of CNTs not only limits the intrinsic magnetic agglomeration leading to homogenized polarization loss, but also enter the interlayers of MXene substrate to form a uniformly dispersed structure resulting in the preformation of a large amount of active surface.

### Analysis of Microwave Absorption Mechanism

**Scheme 2:**
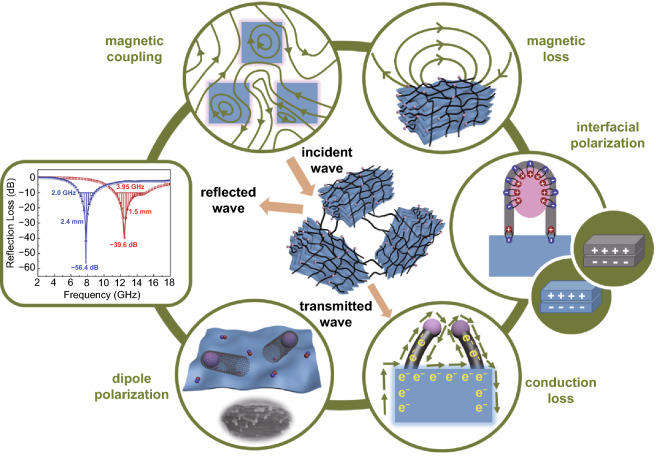
Microwave absorption mechanism of MXene-CNTs/Ni

It is well accepting that EM waves is mainly consumed by the magnetic loss and dielectric loss. The related dissipation mechanisms include the following aspects (Scheme 2).

#### 3D Magnetic Coupling Network by Spatially Mono-dispersed Ni Nanoparticles

**Fig. 6:**
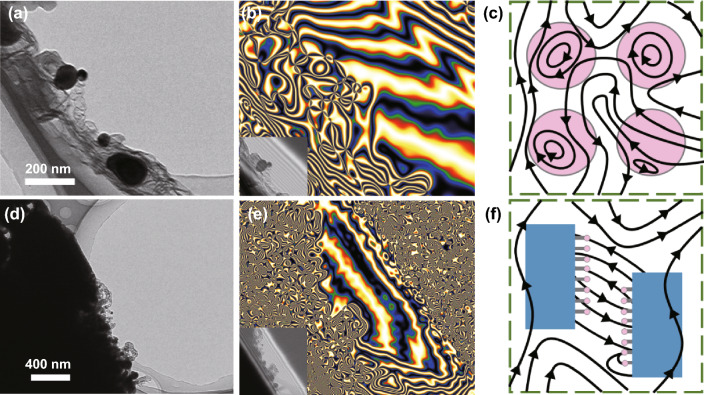
**a** TEM image, **b** corresponding off-axis electron holograms and **c** corresponding magnetic coupling network of CNTs/Ni. **d** TEM figure, **e** corresponding off-axis electron holograms and **f** corresponding magnetic coupling network of MXene-CNTs/Ni

Generally, magnetic nanoparticles are prone to spontaneous aggregation due to their intrinsic magnetism interaction, decreasing magnetic moment intensity around the massive outside surfaces. Moreover, the non-uniformity of the particle size causes the local distribution imbalance, resulting in the instability of the MA performance. While such thorny problems can be effectively solved by our novel 3D architecture. As a dielectric isolator, MXene can separate CNTs that are easy to bend and wind. The bamboo-like CNTs with massive connection joints can robustly support and separate the embedded Ni nanoparticles. Moreover, the confinement effect of the hollow CNTs reasonably regulate the optimized size of the Ni nanoparticles. The Ni nanoparticles with spatial high-density distribution are availably isolated rather than compactly aggregated within each accordion-like MXene unit (Fig. 2d–f), which facilitates the maximized use of magnetic nanoparticles and boots the enhancement of magnetic loss capacity. Each Ni nanoparticle emits magnetic flux lines that can unhinderedly pass across the dielectric carbon walls, proving by the advanced off-axis electron holography (Fig. 6a, b) [44]. The merged magnetic flux lines that composed of neighboring Ni indicate the remarkable magnetic coupling mechanism (Fig. 6c). Benefiting from the existence of 3D coupling network of spatially high-dispersed Ni nanoparticles, each individual MXene unit exhibits the characteristic of being magnetized. Adjacent magnetized MXene units emit out high density of magnetic flux lines to form coupling network at micro-scale, which is considerably exceeds that of traditionally nano-scale size reported (Fig. 6d–f). Such strong magnetic induction signals can intensively interact with the incident EM wave, increasing the magnetic loss capability. Compared with the previously MXene-based simplified structures, the weak magnetic loss behaviors of the local aggregated magnetic nanoparticles greatly reduce their MA performance. By contrast, our novel structure effectively utilizes the gaps between layers of MXene and the confinement effect of CNTs, exposing fully the outer surface with intrinsic magnetic moment of the Ni nanoparticles. Ni nanoparticles are not directly decorated in the MXene unit, while are closely around the 2D MXene through the oriented 1D CNTs (Fig. 3h). Such 3D magnetic coupling behavior with increased magnetic loss capability can rapidly consume EM waves.

#### Enhanced Dielectric Polarization by Multiple Interfaces

**Fig. 7:**
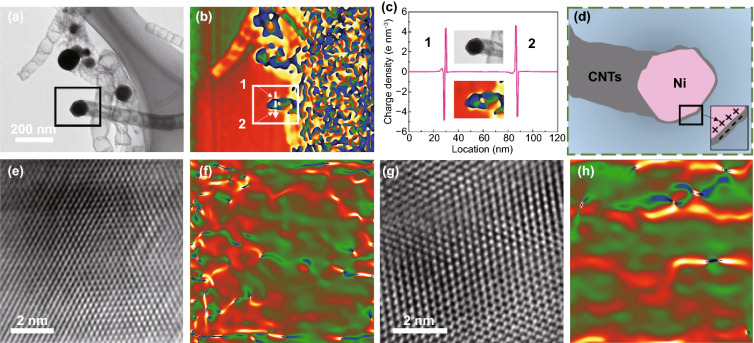
**a** TEM image, **b** charge density map, **c** the profile of charge density in the region, **d** corresponding equivalent model, **e** HRTEM images and **f** corresponding strain maps of the Ni nanoparticles, **g** HRTEM images and **h** corresponding strain maps of the MXene nanosheets. The color scale of **e, g** is black (− 0.5) to white (+ 0.5)

The dielectric loss capability depends mainly on the interfacial polarization and dipolar polarization. The charge density in interfacial region can be evaluated by the Poisson’s equation [45]

4 $$\rho \left( \chi \right) = - \varepsilon _{\gamma } \varepsilon _{0} \frac{{\partial ^{2} \nu \left( \chi \right)}}{{\partial \chi ^{2} }} ,$$

where *χ* is the distance, *ρ*(*χ*) is the charge density, respectively. Some electron (negative) and charge (positive) are enriched near the connect joints of a single carbon tube (Fig. S9). Figure 7b is the charge density distribution diagram. The area indicated by the white arrow extends from the upper side of the CNTs to the Ni nanoparticles and further to the lower side of the surrounding carbon shell. The value of corresponding charge density is from negative to positive, especially a large number of carriers are gathered at the intersection of CNTs and Ni. The enrichment intensity of these carriers far exceeds their enrichment intensity at the carbon tube node, indicating that the interfacial polarization loss is enhanced. Previous studies have confirmed that there is an interface polarization phenomenon in which carriers accumulate at the adjacent layers of MXene. Under the external EM field, multiple carriers can quickly migrate and accumulate around the heterogeneous and homogeneous interface. The unbalance charge distribution and the associated build-in electric field between Ni and CNTs in each 3D MXene unit promote the strong interfacial polarization loss. Compared with the binary compounds, ternary composites greatly increase the number of the heterojunction interfaces. The hopping of electrons around three substances perfectly avoids the problem of local imbalanced matching caused by the only single component.

Massive defects are created in the Ni nanoparticles during the process of catalytic CNTs generation, correlating well with corresponding geometric phase analysis (GPA) map (Fig. 7e, f) [46, 47]. After a strong etching process, some oxygen-containing functional groups inevitably appear on the surface of MXene. There are many points with reversal color appearing in Fig. 7h using the stress–strain analysis of GPA, corresponding to the typical dislocation center. Such points can be regarded as the dipole active sites. When an electron passes through these locations, its geometric center could shift and resulting in the strong dipole polarization. Thus, various polarization and its related relaxation caused by the formation of in situ CNTs/Ni in MXene substrate could dissipate a lots of incident EM wave. More importantly, CNTs are uniformly dispersed on the each MXene unit, bridging the adjacent MXene together to construct an overall network. The bridging effect of CNTs with high conductivity provide more conductive paths for electrons migration and transition, which is conducive to the formation of the 3D conductive network.

#### Well-Matched Impedance Condition by Reasonable Structure Design

**Fig. 8:**
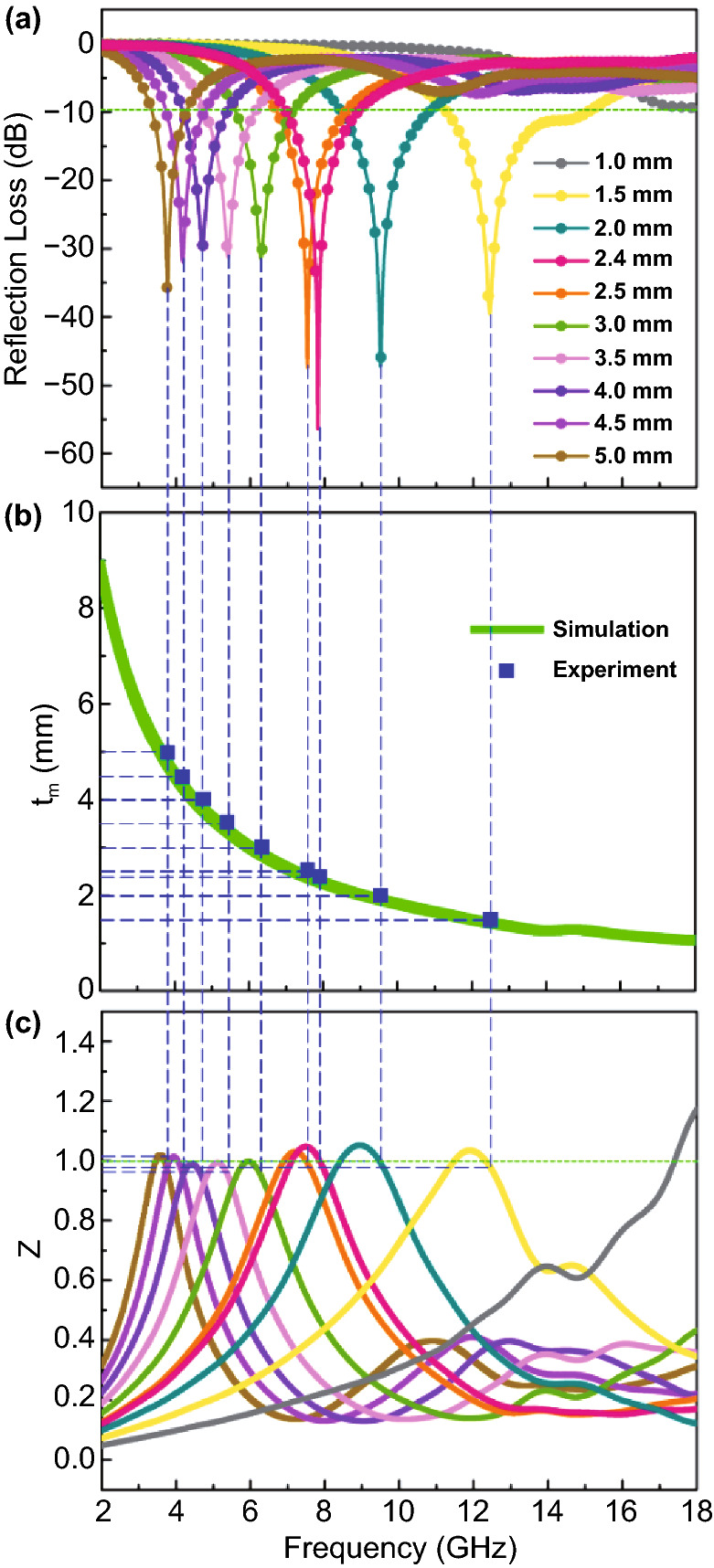
**a** RL-frequency curves, **b** relationship between simulation thickness and peak frequency, and **c** relationship between *Z*_in_/*Z*_0_ and electromagnetic wave frequency of MXene-CNTs/Ni

As mentioned before, well-matched *Z* value is the precondition for the dissipation of the subsequent incident EM waves (Fig. S7). In order to have a zero reflection at the air-absorber interface, the impedance condition of the absorber (*Z*_in_) should equal that of the free space (*Z*_0_) (*Z* = 1) [48, 49]. The frequency dependence of RL values and *Z* values for MXene-CNTs/Ni are plotted in Fig. 8a, c, respectively. Obviously, the *Z* values corresponding to almost all peaks are infinitely close to 1. The incident EM wave can enter into the MXene-CNTs/Ni hybrids to be further consumed rather than reflect at the air–absorber interface. The CNTs not only improves the low conductivity loss of the multi-layered MXene itself, but also supports the growth of Ni nanoparticles to increase the magnetic loss of the composites. Each MXene unit is wrapped in the loose and porous structure, providing enlarged active sites for incident microwave to dissipate. As shown in Fig. 8b, the blue symbols (thickness corresponding to the minimum RL value) are almost located around the λ/4 curve (the green line, *t*_*m*_), indicating that more EM waves are being consumed.

## Conclusion

In summary, the novel 3D MXene including in situ wrapped CNTs/Ni are firstly fabricated to tackle the EM pollution with distinct MA performance. The double agglomeration issue caused by common magnetic nanoparticles and MXene’s self-stacking are successfully avoided by the intercalated CNTs that embedded the spatial high-dispersion Ni nanoparticles. The RL of MXene-CNTs/Ni achieves as much as − 56.4 dB at only 2.4 mm, proving the effectuality of the introduction of CNTs/Ni as a dielectric/magnetic modifier into MXene to reasonably optimize its impedance matching condition. Such regulated EM parameters benefit from the advantage of ternary architecture: (i) high-density spatial Ni nanoparticles distribution without agglomeration, (ii) increased dielectric loss capability, (iii) ultra-magnetic coupling network at micro-scale. This novel synthesis method could be invaluable in designing other advanced MXene-based composites.

## Supplementary Information

Below is the link to the electronic supplementary material.

Supplementary file1 (PDF 1181 KB)
