# Supplementary material for: 3D Seed-Germination-Like MXene with In Situ Growing CNTs/Ni Heterojunction for Enhanced Microwave Absorption via Polarization and Magnetization
Source: Nanomicro Lett. 2021 Jul 19;13:157. doi: 10.1007/s40820-021-00680-w (PMC8289940; doi:10.1007/s40820-021-00680-w)
Supplement: Supplementary file 1 — Supplementary file1 (PDF 1181 KB) [file 40820_2021_680_MOESM1_ESM.pdf]

Supporting Information for

3D Seed-Germination-like MXene with In-Situ Growing CNTs/Ni Heterojunction for Enhanced Microwave Absorption via Polarization and Magnetization

Xiao Li[1, 2], Wenbin You[1], Chunyang Xu[1], Lei Wang[1], Liting Yang[1], Yuesheng Li[1, 2], Renchao Che[1, 2, *]

[1]Laboratory of Advanced Materials, Shanghai Key Lab of Molecular Catalysis and Innovative Materials, Fudan University, Shanghai 200438, P. R. China

[2]Department of Materials Science, Fudan University, Shanghai 200438, P. R. China

*Corresponding author. E-mail: rcche@fudan.edu.cn (Renchao Che)

Supplementary Figures and Tables

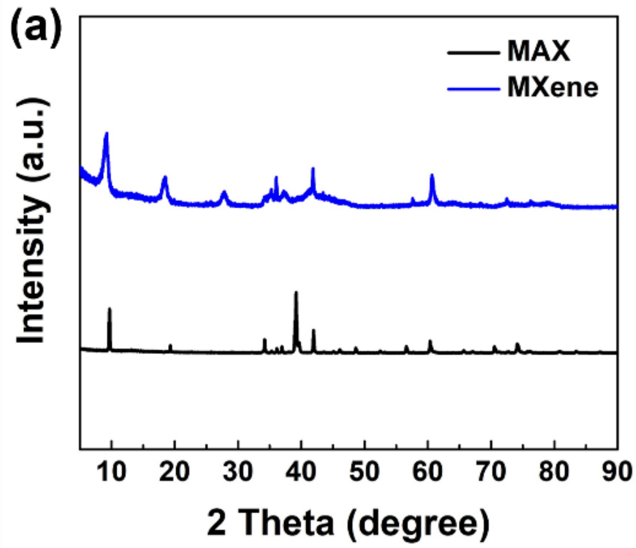

Fig. S1 (**a**) XRD pattern of MAX and MXene

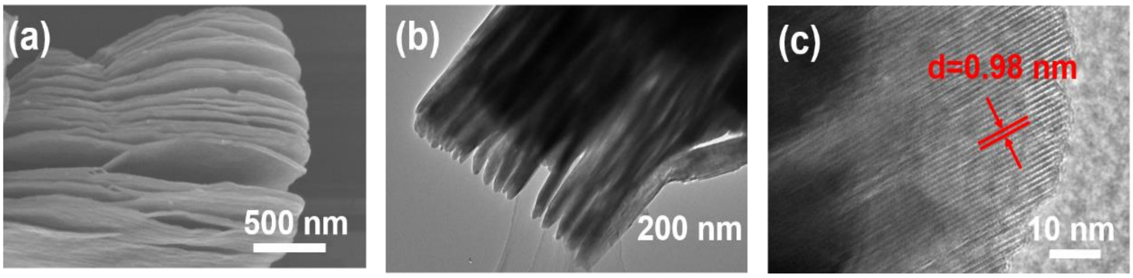

Fig. S2 (**a**) SEM image, (**b**) TEM image and (**c**) HRTEM image of MXene-N

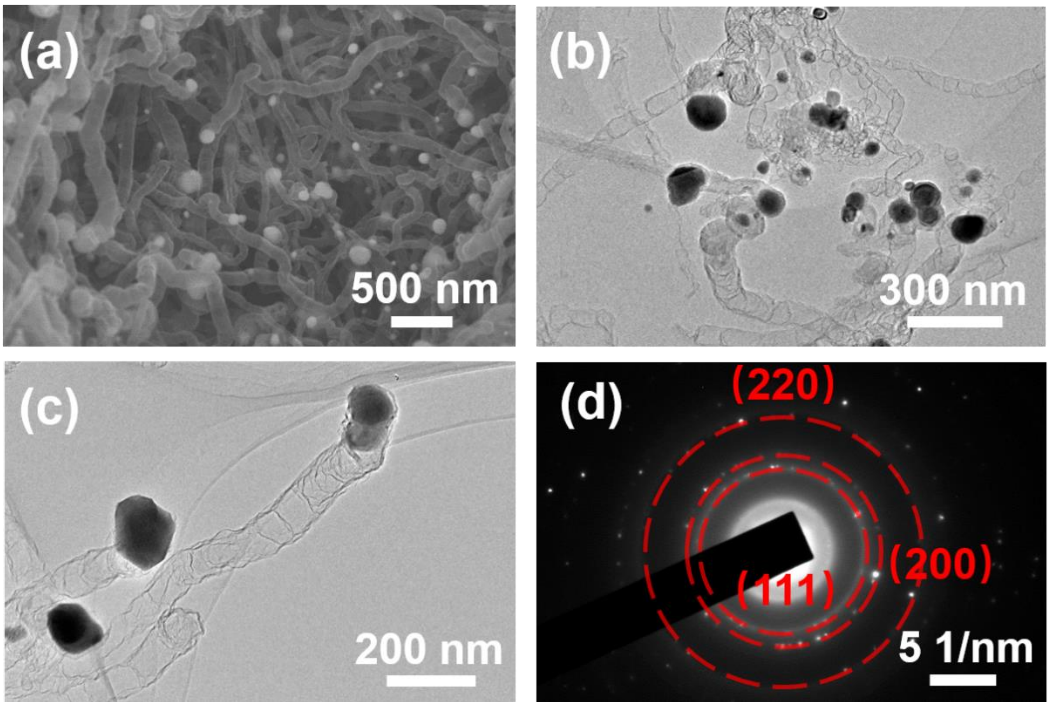

Fig. S3 (**a**) SEM image, (**b**, **c**) TEM image and (**d**) SAED pattern of isolated CNTs/Ni

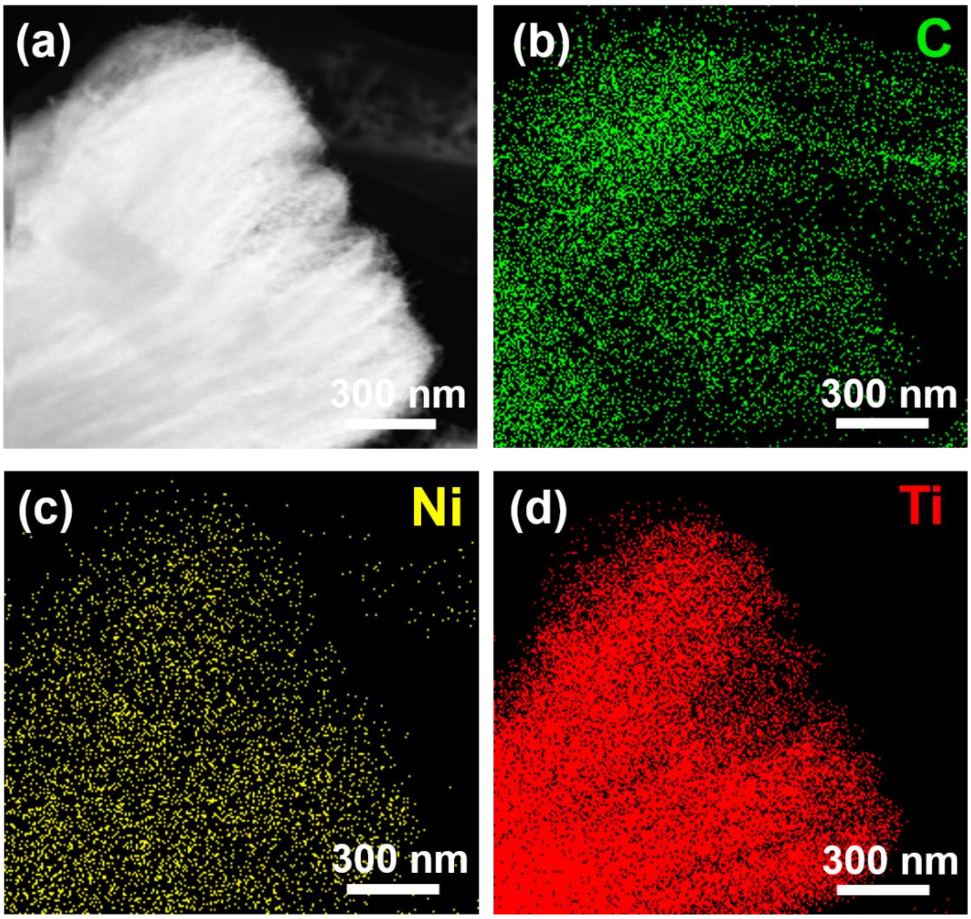

Fig. S4 STEM images of (**a**) Ni^{2+}-MXene-alk and corresponding elemental mapping of (**b**) C, (**c**) Ni and (**d**) Ti

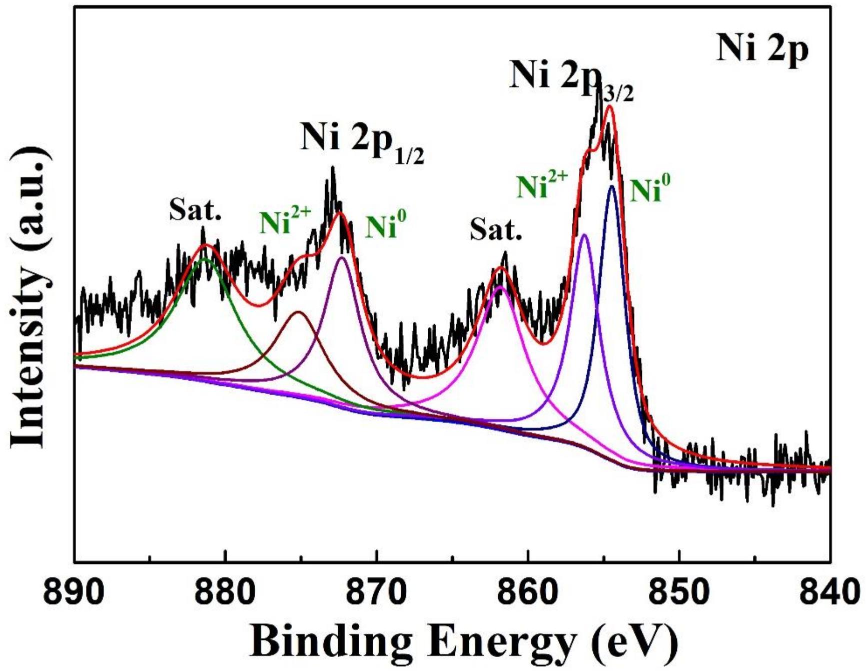

Fig. S5 XPS spectra of Ni 2p in MXene-CNTs/Ni composite

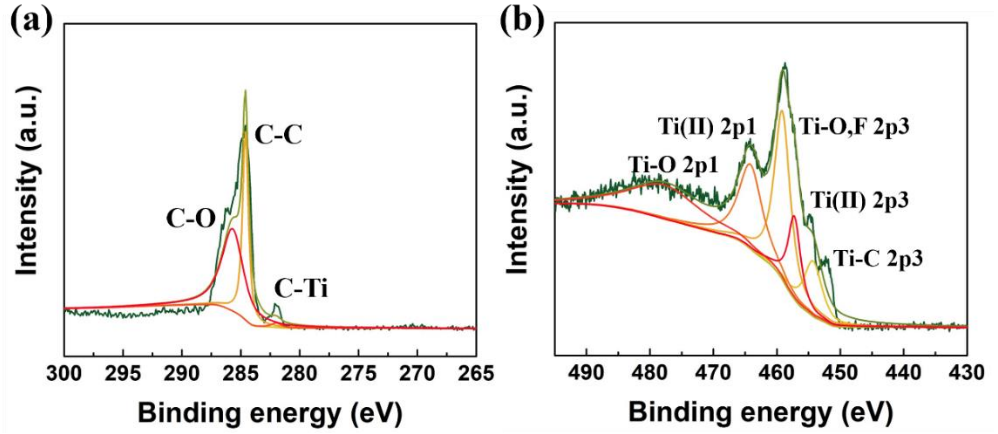

Fig. S6 XPS spectra of C 1s and Ti 2p in MXene-CNTs/Ni composite

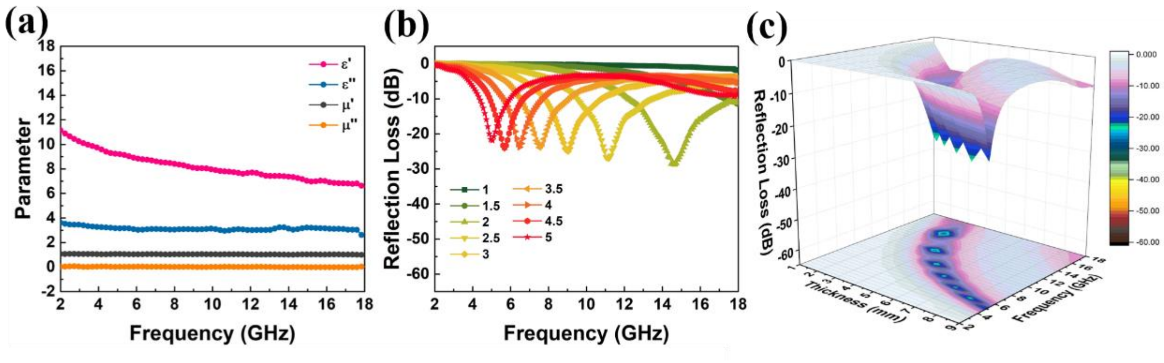

Fig. S7 (**a**) Permittivity and permeability vs frequency, (**b**) RL curves with different thickness and (**c**) 3D plots of MXene/Ni/CNTs composite

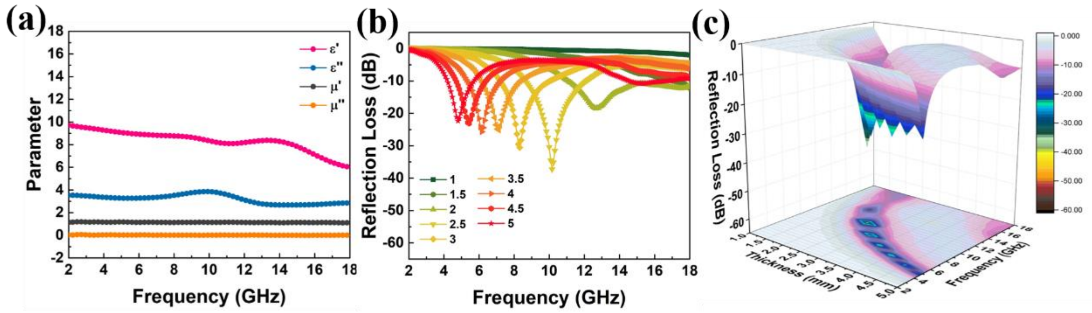

Fig. S8 (**a**) Permittivity and permeability vs frequency, (**b**) RL curves with different thickness and (**c**) 3D plots of MXene/Ni composite

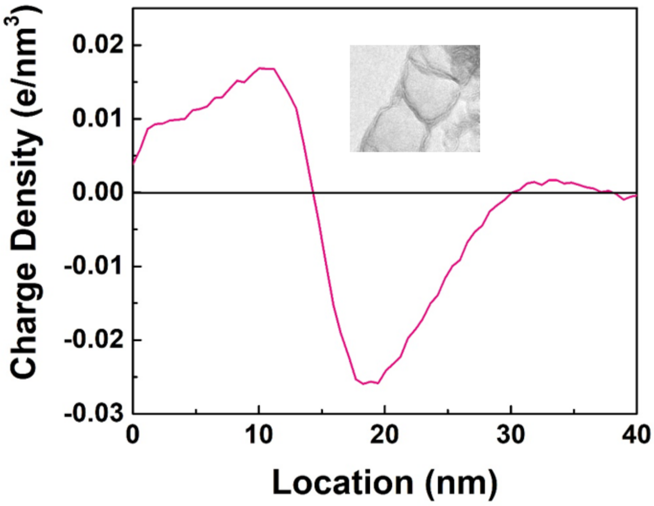

Fig. S9 Profile of charge density in the connect joints of the CNTs

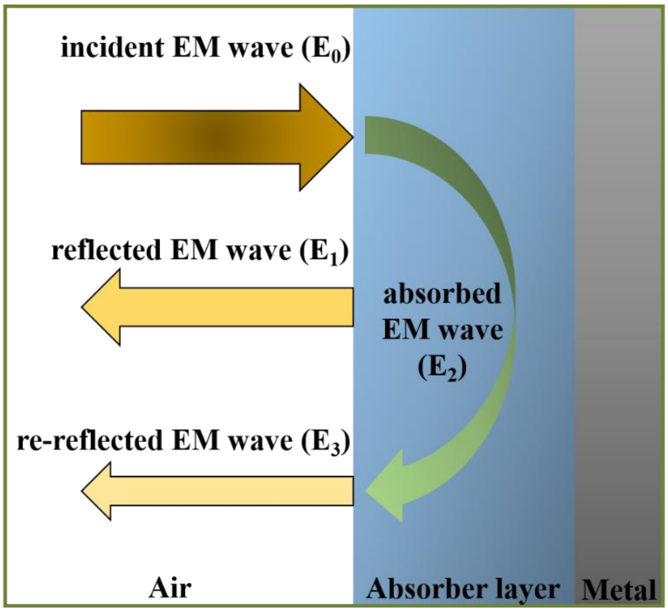

Fig. S10 Microwave absorption model

Table S1 Comparison of MA performance among the reported MXene-based composites and the as-prepared MXene-CNTs/Ni composites

Absorber	RL_{min} (dB)	Matching frequency (GHz)	EAB (GHz)	Thickness (mm)	Refs.
CNTs/Fe	-30.4	3.2	5.76	3.2	[S1]
CNTs/Co	-20.5	2.4	4.08	3.6	[S1]
CNTs/Ni	-34.1	3.2	4.16	3.2	[S1]
MXene/amorphous carbon/TiO_2	-48.4	11.6	2.8	1.85	[S2]
MXene/ZnO	-26.3	17.4	1.4	4	[S3]
MXene/$Ni_{0.5}Zn_{0.5}Fe_2O_4$	-42.5	13.5	3	6.5	[S4]
MXene/PVB/$Ba_3Co_2Fe_{24}O_{41}$	-46.3	5.8	1.6	2.8	[S5]
MXene/Ni-modified	-18.2	16.2	6.3	1.5	[S6]
MXene/Co_3O_4	-34.5	14	6.3	2.0	[S7]
MXene/FeCo	-17.86	-	8.8	1.6	[S8]
MXene/CoFe	-36.29	8.56	2.64	2.2	[S9]
MXene/TiO_2/MoS_2	~ -16	~ 9.8	2.6	2.5	[S10]
MXene/Fe_3O_4/PANI	-40.3	15.3	5.2	1.9	[S11]
MXene/Ni chain	-49.9	11.9	2.1	1.75	[S12]
MXene/carbonyl iron	-15.52	12.8	8.16	1	[S13]
MXene/Ni	-24.3	9.8	2.6	2.2	[S14]
**MXene-CNTs/Ni**	**-56.4**	**7.82**	**3.95**	**2.4**	**this work**

The basic principle of geometric phase analysis (GPA) is as follows:

For a perfect crystal, a HRTEM can be described as a Fourier series:

$$\mathrm{I(r)} = \sum_{g} H_g \, e^{2\pi i g \cdot r}$$

Where I(r) is the image intensity at the position r, g is the periodicities of the Bragg reflections, the Fourier coefficients H_g can be described as:

$$\mathrm{H(g)} = A_g e^{ip_g}$$

Where A_g is the amplitude of the set of sinusoidal lattice fringes g, P_g is the lateral position of the fringes in the original image.

In real image conditions, the Fourier coefficient H_g has conjugate symmetry. Image strength can be expressed as the following real number function:

$$\mathrm{I(r)} = A_0 + \sum_{g>0} 2A_g \cos\left(2\pi g \cdot r + p_g\right)$$

When processing the actually captured high-resolution image, the lattice image is subjected to fast Fourier transform processing to obtain an inverted space bitmap. A specific $\pm$g direction lattice is selected by a mask to obtain a specific direction stripe

information, and then an inverse Fourier transform is performed to obtain a lattice fringe $B_g(r)$ in the specific direction:

$$B_g(r) = 2A_g\cos(2\pi g \cdot r + p_g)$$

In order to describe the lattice changes caused by distortion and defects in the material, the amplitude and phase of the lattice fringes should be expressed by the functions $A_g(r)$ and $P_g(r)$ for the position π, which should be written as:

$$B_g(r) = 2A_g(\mathrm{r})\cos(2\pi g \cdot r + p_g(r))$$

Supplementary References

[S1] M. Ning, J. Li, B. Kuang, C. Wang, D. Su et al., One-step fabrication of N-doped CNTs encapsulating M nanoparticles (M = Fe, Co, Ni) for efficient microwave absorption. Appl. Surface Sci. **447**, 244-253 (2018). https://doi.org/10.1016/j.apsusc.2018.03.242

[S2] M. Han, X. Yin, H. Wu, Z. Hou, C. Song et al., Ti_3C_2 MXenes with modified surface for high-performance electromagnetic absorption and shielding in the X-band. ACS Appl. Mater. Interfaces **8**(32), 21011-21019 (2016). https://doi.org/10.1021/acsami.6b06455

[S3] Y. Qian, H. Wei, J. Dong, Y. Du, X. Fang et al., Fabrication of urchin-like ZnO-MXene nanocomposites for high-performance electromagnetic absorption. Ceram. Int. **43**(14), 10757-10762 (2017). https://doi.org/10.1016/j.ceramint.2017.05.082

[S4] Y. Li, X. Zhou, J. Wang, Q. Deng, M. Li et al., Facile preparation of in situ coated $Ti_3C_2T_x/Ni_{0.5}Zn_{0.5}Fe_2O_4$ composites and their electromagnetic performance. RSC Adv. **7**(40), 24698-24708 (2017). https://doi.org/10.1039/c7ra03402d

[S5] H. Yang, J. Dai, X. Liu, Y. Lin, J. Wang et al., Layered PVB/$Ba_3Co_2Fe_{24}O_{41}/Ti_3C_2$ MXene composite: enhanced electromagnetic wave absorption properties with high impedance match in a wide frequency range. Mater. Chem. Phys. **200**, 179-186 (2017). https://doi.org/10.1016/j.matchemphys.2017.05.057

[S6] W. Feng, H. Luo, S. Zeng, C. Chen, L. Deng et al., Ni-modified Ti_3C_2 MXene with enhanced microwave absorbing ability. Mater. Chem. Front. **2**(12), 2320-2326 (2018). https://doi.org/10.1039/c8qm00436f

[S7] R. Deng, B. Chen, H. Li, K. Zhang, T. Zhang et al., MXene/Co_3O_4 composite material: Stable synthesis and its enhanced broadband microwave absorption. Appl. Surface Sci. **488**, 921-930 (2019). https://doi.org/10.1016/j.apsusc.2019.05.058

[S8] J. He, D. Shan, S. Yan, H. Luo, C. Cao et al., Magnetic FeCo nanoparticles-

decorated Ti_3C_2 MXene with enhanced microwave absorption performance. J. Magn. Magn. Mater. **492**, (2019). https://doi.org/10.1016/j.jmmm.2019.165639

[S9] C. Zhou, X. Wang, H. Luo, L. Deng, S. Wang et al., Interfacial design of sandwich-like CoFe@$Ti_3C_2T_x$ composites as high efficient microwave absorption materials. Appl. Surface Sci. **494**, 540-550 (2019). https://doi.org/10.1016/j.apsusc.2019.07.208

[S10] H. Wang, H. Ma. The electromagnetic and microwave absorbing properties of MoS_2 modified $Ti_3C_2T_x$ nanocomposites. J. Mater. Sci-Mater. Electron. **30**(16), 15250-15256 (2019). https://doi.org/10.1007/s10854-019-01897-7

[S11] Y. Wang, X. Gao, L. Zhang, X. Wu, Q. Wang et al., Synthesis of Ti_3C_2/Fe_3O_4/PANI hierarchical architecture composite as an efficient wide-band electromagnetic absorber. Appl. Surface Sci. **480**, 830-838 (2019). https://doi.org/10.1016/j.apsusc.2019.03.049

[S12] L. Liang, G. Han, Y. Li, B. Zhao, B. Zhou et al., Promising $Ti_3C_2T_x$ MXene/Ni chain hybrid with excellent electromagnetic wave absorption and shielding capacity. ACS Appl Mater Interfaces **11**(28), 25399-25409 (2019). https://doi.org/10.1021/acsami.9b07294

[S13] S. Yan, C. Cao, J. He, L. He, Z. Qu. Investigation on the electromagnetic and broadband microwave absorption properties of Ti_3C_2 MXene/flaky carbonyl iron composites. J. Mater. Sci-Mater. Electron. **30**(7), 6537-6543 (2019). https://doi.org/10.1007/s10854-019-00959-0

[S14] Y. Liu, S. Zhang, X. Su, J. Xu, Y. Li, Enhanced microwave absorption properties of Ti_3C_2 MXene powders decorated with Ni particles. J. Mater. Sci. **55**(24), 10339-10350 (2020). https://doi.org/10.1007/s10853-020-04739-8
